# Impact of 3D-Printed Anatomical Models on Doctor-Patient Communication in Orthopedic Consultations: A Randomized Clinical Trial

**DOI:** 10.7759/cureus.70822

**Published:** 2024-10-04

**Authors:** Alessandro U Sanchez, Guilherme Q Dos Anjos, Diego Gabriel C de Oliveira, Jairo de Andrade Lima, Marina S de Lira, Mário A Costa Júnior, Mucio Brandão V de Almeida, Rodrigo M Heilmann, Epitácio L Rolim Filho

**Affiliations:** 1 Medical Science Center, Federal University of Pernambuco, Recife, BRA; 2 Department of Orthopedics and Traumatology, Clinics Hospital of Federal University of Pernambuco, Recife, BRA

**Keywords:** anatomical models, health communication, orthopedics, patient education as topic, physician-patient relations, three-dimensional (3d) printing

## Abstract

Effective communication between doctors and patients is essential for treatment adherence and better clinical outcomes. Although 3D printing has advanced in medicine, its impact on doctor-patient communication still requires further investigation. This randomized clinical trial evaluated the effectiveness of 3D anatomical models as a tool to facilitate communication in orthopedic consultations. This randomized clinical trial was conducted between May 2024 and September 2024, with 46 patients randomized into two groups: 21 patients received medical explanations with the aid of 3D models, and 25 without. Patients' knowledge was assessed before and after the consultation, and the quality of communication was measured using the Communication Assessment Tool (CAT). In the group using 3D models, 76.19% of patients reported improved knowledge of their conditions, while in the group without models, the increase was 52.00%. Additionally, 14 out of 15 CAT parameters showed statistically significant differences between the groups, with p-values ranging from 0.001 to 0.021. The use of 3D models significantly improved patients' understanding and facilitated communication with doctors, proving to be an effective tool for explaining complex medical conditions.

## Introduction

Effective communication is a fundamental skill in medical practice and plays a crucial role in various aspects of healthcare. Good communication not only increases patient satisfaction with the healthcare team but also has direct effects on clinical outcomes. Studies show that effective communication contributes to better patient recovery after surgical procedures, promotes more balanced emotional health after discharge, and encourages greater adherence to prescribed treatment, resulting in better clinical outcomes and improved quality of life for patients [1-4]. Moreover, the positive impact is not limited to patients; healthcare professionals also benefit, experiencing increased empathy, enhanced self-efficacy, and a significant reduction in burnout rates, which is a growing problem among healthcare workers [5].

The doctor-patient relationship, in addition to its emotional importance, can influence practical factors such as the length of hospital stay and the number of clinical complications during treatment, which directly affect treatment costs. This financial impact underscores the need to improve doctors' communication skills to optimize healthcare resources and ensure more efficient treatments [6].

However, communication between doctors and patients faces several challenges. One of the main problems is the difficulty many professionals encounter when explaining complex procedures, especially those involving anatomical structures that are difficult to visualize. In surgical situations, this communication barrier can increase patient anxiety and reduce understanding of the risks and benefits of the proposed treatments [7].

An innovative solution that has been adopted by medical teams to facilitate this communication is the use of anatomical models printed in 3D. This technology allows for a clearer visualization of the affected structures, aiding in the explanation of complex clinical conditions and in demonstrating necessary procedures [8,9]. Although the first patents related to 3D printing date back to the 1970s, it was only after 2010, with the expiration of several patents, that the technology became popular and began to be widely used in medicine [10].

Currently, the application of 3D printing in medicine is rapidly expanding, benefiting both surgeons and patients. Its main applications in healthcare include surgical planning, the creation of customized orthoses, the development of surgical guides, and even the production of patient-specific implants. Additionally, these models are valuable tools for medical education, allowing students and residents to train with realistic representations of anatomical structures [11]. Despite advances in the use of 3D printing, most studies focus on its role in medical training [12] and surgical planning [13], while the impact of this technology on doctor-patient communication still requires further investigation.

A well-established way to assess the quality of medical communication is through the Communication Assessment Tool (CAT), which consists of a 15-item survey focused on basic communication skills. In this evaluation, patients are asked about their specific experience with the doctor, providing detailed and specific feedback that can help professionals improve their communication performance [14,15].

Given this context, the present study aims to evaluate the effectiveness of using 3D anatomical models created by 3D printers as a tool to facilitate communication between doctors and patients in an orthopedic outpatient setting. It is expected that the use of these models will contribute to a better understanding by patients of their health conditions, increase satisfaction with care, and potentially positively influence clinical outcomes.

## Materials and methods

This is a randomized clinical trial conducted in the Orthopedics and Traumatology outpatient clinic of the Hospital das Clínicas of the Federal University of Pernambuco (HC-UFPE) in Pernambuco, Brazil, between May 2024 and September 2024. The study was approved by the Research Ethics Committee of the Health Sciences Center at the Federal University of Pernambuco (CAAE: 74428223.2.0000.5208), and all participants provided written informed consent.

Patients aged 18 years or older who underwent consultations or procedures in the Orthopedics and Traumatology department were included. Patients without available radiological exams, with systemic conditions associated with the orthopedic clinical picture, without a confirmed orthopedic diagnosis, or with pathologies not suitable for explanation through 3D models were excluded.

The participants were randomly assigned by simple draw into two groups: the group that received explanations with the aid of 3D-printed anatomical models, and the group that received explanations without the models using only imaging exams (X-rays, CT scans, or MRIs). In the group with 3D models, patients received detailed explanations about the macroscopic anatomical changes related to their diagnosis, treatment options, and prognosis. The group without 3D models received the same information but based solely on the imaging exams.

After the consultation, all patients completed a questionnaire (presented in Appendices) that collected demographic data, such as their level of education, and included a self-assessment of their knowledge about their condition before and after the explanation. Patients were then categorized into those who increased their self-assessment of knowledge and those who did not. Additionally, the CAT was used consisting of 14 questions that evaluate patients' perception of doctor-patient communication during the consultation and one final question to assess the patient’s overall perception of the doctor responsible for the care.

Data were stored using Microsoft Excel 2019 software (Microsoft Corp., Redmond, USA), and statistical analysis was performed using IBM SPSS software version 21 (IBM Corp., Armonk, USA). Nominal variables were described in absolute and relative frequencies, while quantitative variables were described as means and standard deviations. To compare the distribution between categorical variables, the chi-square test was used, and for the analysis of ordinal variables, such as CAT evaluations, the Mann-Whitney U test was applied.

## Results

In total, 46 patients were evaluated from the outpatient clinic of the Orthopedics and Traumatology department at HC-UFPE. Patients had a mean age of 60.43 years with a standard deviation of 11.65 years. The group whose explanation occurred with 3D printing had 21 patients, while the group without 3D printing had 25 patients. Information on the absolute and relative frequencies of participants' education and gender are presented in Table 1.

**Table 1:** Demographic data of participants

Demographic Data of Participants						
Variable		With 3D Printed Model		Without 3D Printed Model		p-value
		N	% of 46	N	% of 46	
Sex						p=0.106
	Male	9	19.57%	12	26.09%	
	Female	12	26.9%	13	28.26%	
Education level						
	Incomplete elementary school	13	28.26%	8	17.39%	p=0.069
	Complete elementary school	3	6.52%	2	4.35%	
	Incomplete high school	2	4.35%	1	2.17%	
	Complete high school	2	4.35%	7	15.22%	
	Completed higher education	1	2.17%	7	15.22%	

When asked about their knowledge of the condition that affected them before and after the consultation, most patients were inclined to say that they assessed their knowledge before the consultation as little or none. But, after the explanations, both the group with 3D printing and the group without 3D printing were inclined to evaluate the knowledge as good or very good. Comparing knowledge assessments before and after the consultation, it is clear that 76.19% of individuals in the group assisted with a 3D model improved their assessment, while in the group without 3D models, an improvement in assessment was noted in 52% of the participants. Information regarding the evaluation and comparison of the improvement in the evaluation profile of each group is presented in Table 2.

**Table 2:** Assessment of knowledge before and after explanation

Assessment of Knowledge Before and After Explanation								
Group		None	Little	Good	Extensive	Improved Evaluation		p-value
						Yes	No	
With 3D printed model								
	Before	6	11	4	0	16	5	p=0.082
	After	0	1	17	3			
Without 3D printed model								
	Before	4	11	8	2	13	12	
	After	1	2	20	2			

When evaluated by the CAT, in most questions, both groups were inclined to evaluate the question as excellent, while no patient evaluated the questions as poor or far. The responses to the questionnaires from both groups, as well as the comparison between the assessments, are shown in Table 3.

**Table 3:** Communication Assessment Tool comparison by group

Communication Assessment Tool Comparison by Group							
Question	Group	Poor	Fair	Good	Very Good	Excellent	p-value
1. Greeted me in a way that made me feel comfortable							
	With 3D printed model	0	0	0	2	19	p=0.019
	Without 3D printed model	0	0	1	9	15	
2. Treated me with respect							
	With 3D printed model	0	0	0	1	20	p=0.021
	Without 3D printed model	0	0	1	7	17	
3. Showed interest in my ideas about my health							
	With 3D printed model	0	0	0	2	19	p=0.019
	Without 3D printed model	0	0	1	9	15	
4. Understood my main health concerns							
	With 3D printed model	0	0	0	3	18	p=0.052
	Without 3D printed model	0	0	1	9	15	
5. Paid attention to me (looked at me, listened carefully)							
	With 3D printed model	0	0	0	2	19	p=0.019
	Without 3D printed model	0	0	1	9	15	
6. Let me talk without interruptions							
	With 3D printed model	0	0	0	2	19	p=0.002
	Without 3D printed model	0	0	1	12	12	
7. Gave me as much information as I wanted							
	With 3D printed model	0	0	0	2	19	p=0.005
	Without 3D printed model	0	0	1	11	13	
8. Talked in terms I could understand							
	With 3D printed model	0	0	0	3	18	p=0.001
	Without 3D printed model	0	0	1	15	9	
9. Checked to be sure I understood everything							
	With 3D printed model	0	0	0	2	19	p=0.010
	Without 3D printed model	0	0	1	10	14	
10. Encouraged me to ask questions							
	With 3D printed model	0	0	0	3	18	p=0.003
	Without 3D printed model	0	0	2	12	11	
11. Involved me in decisions as much as I wanted							
	With 3D printed model	0	0	0	3	18	p=0.002
	Without 3D printed model	0	0	1	14	10	
12. Discussed next steps, including any follow-up plans							
	With 3D printed model	0	0	0	2	19	p=0.005
	Without 3D printed model	0	0	1	11	13	
13. Showed care and concern							
	With 3D printed model	0	0	0	2	19	p=0.010
	Without 3D printed model	0	0	1	10	14	
14. Spent the right amount of time with me							
	With 3D printed model	0	0	0	2	19	p=0.010
	Without 3D printed model	0	0	1	10	14	
15. The responsible team treated me with respect							
	With 3D printed model	0	0	0	1	20	p=0.006
	Without 3D printed model	0	0	1	9	15	

## Discussion

The study results showed that there was no statistically significant difference in the demographic data of the patients from the analyzed groups. There was a slight predominance of women and a higher concentration of patients with low education levels, which reflects the profile of the orthopedic and trauma outpatient clinic at HC-UFPE, a public hospital in the Northeastern region of Brazil. This pattern is consistent with other studies indicating a predominance of patients with lower educational levels among users of the public health system in Brazil [16,17].

When assessing patients' prior knowledge about their conditions before the medical explanation, most reported having little to no understanding of their pathology. This aligns with the study by Waryasz et al. [18], which found that less than half of orthopedic patients knew which bone they had fractured, and an even smaller percentage understood the expected healing time. The same study also pointed out a lower understanding among patients with lower educational levels, a characteristic that matches the profile of the participants in our study. Cosic et al. [19] reinforce this observation by comparing the level of medical knowledge between patients in public and private hospitals.

Although the improvement in knowledge self-perception was more pronounced in the group that used 3D models (76.19%) compared to the control group (52%), this difference was not statistically significant (p=0.082). However, the observed trend suggests a potential benefit of using 3D models, supporting findings from previous studies. Hong et al. [7] demonstrated that these models enhance the understanding of patients undergoing thyroid procedures, while Samaila et al. [20] reported that the use of 3D models increased the confidence of trauma patients in their treatments. Nevertheless, methodological differences between the studies should be considered: Hong et al. assessed patients' understanding of thyroid anatomy, which is relatively more complex than musculoskeletal anatomy, while Samaila et al. focused on patients with specific traumas, which may have contributed to the variation in results. These factors highlight the need for further investigations to determine how 3D models can be effective in different clinical settings and populations.

The CAT assessment indicated that patients who received explanations with a 3D model had a more positive perception of communication with the medical team, corroborating findings from studies such as Yang et al. [21], which highlights that 3D printing, by faithfully replicating fracture anatomy, facilitates communication between doctor and patient. Zheng et al. [22] also reported that patients view the use of 3D models as a positive tool to improve communication, and medical professionals also evaluate this technology positively [22,23].

Thus, it is evident that 3D printing has a relevant impact on improving communication between doctors and patients, which may contribute to better recovery, greater adherence to treatments, and improved clinical outcomes. It is imperative that further studies be conducted to evaluate the longitudinal effects of this tool on patient health outcomes.

Although it is an increasingly accessible technology, healthcare professionals must be aware of the potential of 3D printing as a cost-effective and efficient tool to improve communication and patient prognoses.

Limitations

This study presents some limitations that should be considered. First, the sample consisted of patients from an orthopedic and trauma outpatient clinic, without distinction between orthopedic subspecialties. Different pathologies or anatomical complexities may influence patients' perceptions of the use of 3D models, suggesting the need for more specific studies. Additionally, the relatively small sample size (n=46) may have limited the statistical power of the study, reducing the ability to detect significant differences even when trends were observed. Larger samples in future studies could provide a more robust assessment of the impact of 3D models. Finally, the study only evaluated outcomes related to medical communication immediately after the consultation, which limits the analysis of the long-term impacts of this approach.

## Conclusions

The findings of this study demonstrate that the use of 3D-printed anatomical models during medical explanations was effective in improving understanding and communication between doctors and patients in an orthopedic outpatient clinic. Patients who received explanations with the aid of 3D models reported a more positive perception of their understanding of their conditions and rated the communication with the medical team more highly compared to the group that did not use the models. These results suggest that the use of 3D printing can be a valuable tool to facilitate the explanation of complex conditions and improve the patient experience in the clinical setting.

## Appendices

Date:________________________________ Name:_______________________________

Demographic data

Age:_________                           Sex: (   ) Male  (   ) Female  (   ) Other/I don't want to respond

Education level: (   ) Incomplete Elementary School                   (   ) Complete Elementary School (   ) Incomplete High School                                (   ) Complete High School (   ) Incomplete Higher Education                      (   ) Completed Higher Education

Assessment of knowledge before and after explanation

How do you self-assess your knowledge about the disease before the consultation? (   ) None knowledge          (   ) Little knowledge           (   ) Good knowledge         (   ) Extensive knowledge

How do you self-assess your knowledge about the disease after the consultation? (   ) None knowledge          (   ) Little knowledge           (   ) Good knowledge         (   ) Extensive knowledge

Communication Assessment Tool (CAT)

Communication with patients is a very important part of quality medical care. We would like to know how you feel about the way your doctor communicated with you. Your answers are completely confidential, so please be as open and honest as you can. Thank you very much. 1. Poor          2. Fair          3. Good          4. Very good          5. Excellent

Please use this scale to rate the way the doctor communicated with you:

The doctor... 1. Greeted me in a way that made me feel comfortable...................................1          2         3         4         5 2. Treated me with respect.............................................................1          2         3         4         5 3. Showed interest in my ideas about my health.........................................1          2         3         4         5 4. Understood my main health concerns..................................................1          2         3         4         5 5. Paid attention to me (looked at me, listened carefully).............................1          2         3         4         5 6. Let me talk without interruptions...................................................1          2         3         4         5 7. Gave me as much information as I wanted.............................................1          2         3         4         5 8. Talked in terms I could understand..................................................1          2         3         4         5 9. Checked to be sure I understood everything..........................................1          2         3         4         5 10. Encouraged me to ask questions.....................................................1          2         3         4         5 11. Involved me in decisions as much as I want.........................................1          2         3         4         5 12. Discussed next steps, including any follow-up plans................................1          2         3         4         5 13. Showed care and concern............................................................1          2         3         4         5 14. Spent the right amount of time with me.............................................1          2         3         4         5

The doctor's staff... 15. Treated me with respect............................................................1          2         3         4         5
